# Qualified and Unqualified (N-R C) mental health nursing staff - minor differences in sources of stress and burnout. A European multi-centre study

**DOI:** 10.1186/1472-6963-10-163

**Published:** 2010-06-14

**Authors:** Knut W Sorgaard, Peter Ryan, Ian Dawson

**Affiliations:** 1 Nordland Hospital Trust, 8092 Bodo, Norway/Institute of Clinical Medicine, University of Tromso, Norway; 2 Peter Ryan, Middlesex University, Archway Campus, Archway, N195NFL London, UK; 3 Ian Dawson, Nordland Hospital Trust, 8092 Bodo, Norway

## Abstract

**Background:**

Unqualified/non-registered caregivers (N-R Cs) will continue to play important roles in the mental health services. This study compares levels of burnout and sources of stress among qualified and N-R Cs working in acute mental health care.

**Methods:**

A total of 196 nursing staff - 124 qualified staff (mainly nurses) and 72 N-R Cs with a variety of different educational backgrounds - working in acute wards or community mental teams from 5 European countries filled out the Maslach Burnout Inventory (MBI), the Mental Health Professional Scale (MHPSS) and the Psychosocial Work Environment and Stress Questionnaire (PWSQ).

**Results:**

(a) The univariate differences were generally small and restricted to a few variables. Only Social relations (N-R Cs being less satisfied) at Work demands (nurses reporting higher demands) were different at the .05 level. (b) The absolute scores both groups was highest on variables that measured feelings of not being able to influence a work situation characterised by great demands and insufficient resources. Routines and educational programs for dealing with stress should be available on a routine basis. (c) Multivariate analyses identified three extreme groups: (i) a small group dominated by unqualified staff with high depersonalization, (ii) a large group that was low on depersonalisation and high on work demands with a majority of qualified staff, and (iii) a small N-R C-dominated group (low depersonalization, low work demands) with high scores on professional self-doubt. In contrast to (ii) the small and N-R C-dominated groups in (i) and (iii) reflected mainly centre-dependent problems.

**Conclusion:**

The differences in burnout and sources of stress between the two groups were generally small. With the exception of high work demands the main differences between the two groups appeared to be centre-dependent. High work demands characterized primarily qualified staff. The main implication of the study is that no special measures addressed towards N-R Cs in general with regard to stress and burnout seem necessary. The results also suggest that centre-specific problems may cause more stress among N-R Cs compared to the qualified staff (e.g. professional self-doubt).

## Background

A combination of demography, restructured health care systems and social values has made lack of nursing personnel a main concern for health care administrators, politicians and the nursing professions. The growing shortage of health care workers has become an international challenge [1] and there is a predicted shortfall of qualified nursing staff in both low and high-income countries [2-4] The trend towards community care and the resultant closure of mental hospitals compel the nursing staff to provide high quality care to more patients, often with less human and other resources available [5,6]. The role of non-registered caregivers (N-R Cs) in the European health services is not well documented.

Many countries lack national work force strategies addressing numbers and qualifications of the mental health staff, including assistant nursing staff [7]. N-R-C staff often delivers a high proportion of direct contact with patients [8] and it is highly likely that they will continue to serve important functions in the health services in the years to come. In the UK this has long been recognised and N-R-C workers have received national recognition [9].

The mental health field has a long and diverse history of use of subprofessionals. In the 1960's and onwards psychotherapy research often dealt with how efficient lay-therapists were compared with professionally trained therapist [10-13]. Although not attracting the same amount of attention, the issue is still debated e.g. [14]. These questions did not attract the same attention in the caring sectors. With the academisation of the nursing profession and the general focus on qualification, unqualified staff was gradually seen as mainly as temporarily employed waiting to be replaced by professional health workers, preferably nurses. As temporary workers they are seldom mentioned in official health plans, and in relation to the present work we were unable to find either WHO or EU documents that dealt thematically with how the subprofessional work force can fit in today's health services, except for intentional statements that an effective mental health workforce needs to include both professional and subprofessional workers with a range of different backgrounds (WHO 2005). Being considered "substitutes" lead to a corresponding lack of knowledge about the real competency of subprofessionals, what they can bring into the services and how they experiences specific stress and strains.

The quality of the working environment is of great importance for the recruitment and retention of both skilled and N-R C staff. This makes it necessary to focus on work-related stressors as a part of the total outcome spectrum in evaluations of the psychiatric health services [15]. A number of studies have shown that the wellbeing of mental health professionals may be adversely affected by a variety of work-related stressors [16-19], and studies suggest that nurses may have high rates of sickness and absence from work due to stress [20,21]. There is uncertainty about how widespread the problems are and some studies have been unable to corroborate these studies [22-28].

With few exceptions e.g.[29,30] N-R Cs have not received much scientific interest, especially in the mental health field. Few studies have addressed sources of stress and burnout among N-R Cs. Jenkins and Elliott [31] compared stressors and burnout among qualified and N-R C staff in acute mental health services. They found that the two groups did not differ significantly in terms of burnout, but that the sources of stress were to some extent different. The main stressor for the qualified workers was lack of adequate staffing, whereas dealing with threatening, difficult and demanding patients was the most prominent source of stress among N-R C staff. It is the purpose therefore of this article to address this imbalance by focusing on the relative impact of stressors of qualified professional staff compared to non-registered care-givers (NRCs).

### General Context

Theories of stress emphasize the interplay of stress sources, reactions to stress and coping [32,33]. Burnout may be a long-term consequence of demanding situations. Burnout is characterized by feelings of being emotionally drained, having negative attitudes towards the recipients of services and reduced self-competence. It may also seriously disrupt the therapeutic relationship between the service provider and client [34,35]. A proper understanding of burnout requires a transactional perspective, covering the interactions of individual, organizational, and social factors [36,37].

Much research has focused on stress and burnout among nurses [38]. Mental health nurses have been less studied than other nurses in other parts of the health system [31]. Although most studies within mental health have addressed out-patient staff, also in-patient wards can be highly stressful [39]. With the change from inpatient to outpatient and community treatment, concerns have been raised that high levels of stress may threaten the quality and stability of community mental health services [40-42].

The stress literature identifies four principal sources of stress in the nursing profession: (i) educational sources, (ii) clinical sources, (iii) personal/social sources [43] in addition to (iv) organisational causes. Examples of clinical sources are excessive work demands and work pressure, work load, problems with meeting the needs of the service recipients, and violent or suicidal patients [44-47]. Personal and social stress sources include lack of autonomy, home-work balance and ambiguity in personal roles, conflicts in personal values, and insufficient support [48-51]. Organizational stressors are exemplified by conflicts with other professions, lack of financial or personnel resources, organizational changes and/or new routines, insufficient salary, poor managerial or supervisory services, etc [52-55].

A transactional perspective implies that elements from different sources interact in complex ways. For example, Janssen et al [44] found that that whereas emotional exhaustion among nurses was related to the total workload, intrinsic work motivation was influenced by how challenging and worthwhile the work was in terms of range of skills, autonomy, opportunities to learn, and social contacts.

Stress sources related to educational background are the principal focus of the present work. They encompass aspects such as professional development, professional identity, lack of recognition and respect, fear of failing and lack of qualifications. [56,42,57] Although academic education and professional qualification are but one aspect of clinical competence (the others being clinical skills and professionals attitudes), the change of nurse teaching from work-based apprenticeship to academic education and the parallel development of increasingly specialized nursing roles [58,59]) contribute to an alteration of what is considered to be necessary qualifications among nursing staff. This may cause additional strain on the substantial proportion of clinically oriented staff who lack formal (nursing) qualifications.

We believe the importance of the present study lies in the focus it has on working conditions of subprofessionals in acute psychiatry. As we have argued above, there is an increasing and probably worldwide lack of nursing staff in the health services and increased use of health care assistants is reported e.g.[30]. Although the evidence on a general level suggests that more use of less qualified staff will not be effective in all situations [60], due to what is said above it is increasingly important to recruit, retain and qualify also subprofessionals, and a condition for this is the quality of their working environments.

Whilst criticizing the general contention of very stressful working conditions in mental heath care, Richard et al [26] argued for multi-site studies of the working environment in mental health care. The present study compares sources of stress and levels of burnout among qualified and N-R C staff working in acute inpatient and community care settings in 5 centres in 4 European countries: 2 from Denmark as well as Great Britain, Norway and Poland. We expected to find differences with regard to sources of stress between qualified and N-R C nursing staff, and as an effect of this also a higher prevalence of burnout among N-R C staff.

## Methods

### Study design

The study was part of an EU funded framework 5 project aimed at assessing levels of stress and burn out and developing effective strategies for dealing with difficult and dangerous patients in inpatient and community acute psychiatric care. This study is described in greater detail in [61,28]. The study comprised 6 centres, but because one of them (Tampere) reported having no N-R C staff, it was excluded from this part of the study.

At each centre, catchment areas were randomly selected from within the total set of catchment areas available in the centres participating in the study. Teams were then randomly chosen from each site, stratified by in-patient versus community teams. In some cases this resulted in all locally available teams entering the study. Within centres, the in-patient and community staff was drawn from within the same catchment areas, thus ensuring that issues around demographic variations in patient populations were addressed.

### Target groups

It is notoriously difficult to define and classify the different health professionals across countries [62,63]. Moffic [64] uses the term "paraprofessionals" to refer to health workers who are not qualified as psychiatrists, psychologists, social workers or nurses, or who are below a master's degree level of education. In the definition of N-R Cs, we follow Moffic's criteria of being below master level of education and not being qualified as psychiatrists, psychologists, social workers or nurses. All participants were employed in acute care, either in community teams or in acute wards. The study sample consisted of a total of 196 staff members, 124 qualified staff, and 72 N-R C staff. The total participation rate in the study was 72 percent. The professional and educational background of the N-R C varied and included for example degrees in philosophy, and art therapy. The biggest categories were health care assistants (N = 20) and enrolled nurses (N = 11; until 2009 based on an employed model with on pre- required training). An overview of the proportions of qualified to N-R C staff at each centre is given in table 1.

**Table 1:** Percentages and numbers of qualified to unqualified nursing staff

Centre	Qualified	Unqualified
Aarhus	63 % (22)	37 % (13)
Bodø	74 % (34)	26 % (12)
Cambridge	61 % (28)	39 % (18)
Storstrom	35 % (14)	65 % (26)
Warsaw	90 % (26)	10 % (3)

Demographic characteristics of the two groups are presented in table 2.

**Table 2:** Demographic characteristics of the qualified and unqualified staff

Variable	Qualified (N = 124)	Unqualified staff (N = 72)
Age (SD) ^1)^	40.8 (10.4)	43.1 (11.0)
Males ^2)^	16.9 %	27.8 %^1)^
Time in current job (yrs./SD)	6.5 (7.6)	9.8 (10.8)
Time in mental health (yrs./SD)	12.3 (8.4)	11.8 (11.7)
Hrs work each week ^3)^	37.0	32.6
Professional qualifications^4)^	100 %	81.7 %
Shift Work	50.8 %	60.6 %
Only employment	79.7 %	77.5 %
Recipient of violence at work^4)^	81.5 %	70.8 %
Training in dealing with violence in current job^5)^	47.2 %	62.5 %
Training in dealing with violence in previous jobs	31.7 %	22.2 %

^1) ^Z - 1.742, p = .08; ^2) ^Chi-square 3.24, p .07; ^3) ^Z - 3.37 p = .001, ^4) ^Chi-square 2.95, p = .08; ^5) ^Chi-square 4.29, p = .04

The qualified staff worked more hours per week, and had more often been recipient of violence from patients. Unqualified staff was older, consisted of more males and fewer had professional qualifications of any kind.

### Procedure

Once the teams were selected for each centre, the site researchers visited all teams included in the study, explained the study aims, policy with respect to confidentiality etc, then handed each staff member an information leaflet about the study, and answered questions. Questionnaires were left on the wards for self-completion and collected by the site researchers at an agreed timescale later. Staff who did not complete the instruments was followed up with a view to encourage completion.

### Measures

The following self-report measures were used. The OSCAR Demographic Questionnaire - ODQ [65], the Maslach Burnout Inventory - MBI [66], the Mental Health Professional Stress Scale (MHPSS) [67] and the Psychosocial Work Environment and Stress Questionnaire - PWSQ [68,69]. The OSCAR demographic questionnaire comprises 105 items covering basic demographics (age, sex, martial status, number of children, other dependencies, education, employment and other work related questions, dealing with violence at work, stress training, stress support etc.). The MBI was chosen as a reliable and valid indication of levels of burnout among mental health professionals amongst the sites. It is a 22-item measure intended to assess three aspects of the burnout syndrome: Emotional Exhaustion (9 items), Depersonalisation (5 items) and Personal Accomplishment (8 items). Each item is scored on a 7-point scale (None to Every day). Reliability and validity are good. The three-factor structure has proved invariant across different occupational groups, and the internal consistencies of the subscales are satisfactory [70,71]. The MBI is widely used in studies of mental health professionals (usually nursing staff). To identify and measure the causes of stress pertinent to the different professional groups the Mental Health Professionals Scale (MHPSS) was chosen. The MHPSS is a 42-item discipline-neutral scale for identifying sources of stress in mental health professionals. It is suitable for multi-disciplinary work, addresses home-work conflicts and is psychometrically robust. It is grouped into seven scales: Workload, Client-related difficulties, Organisational structure and processes, Relationships and Conflicts with other professionals, Lack of resources, Professional self-doubt and home-work conflict. It is scored on a 4-point scale (from "Does not apply to me" to "Does apply to me"). The scale has good reliability and validity and there is some support for the original factor structure [72].

The social climate or environment is an important source of burnout related stress in a number of previous studies. It was studied by using The Psychosocial Work Environment and Stress Questionnaire (PWSQ). The majority of items are scored as Yes or No. The scale has been thoroughly tested for reliability and validity (Rasch item analyses model) and consists of 10 scales (Job demands, Work load, Job control, Influence, Management style, Role clarity, Social contact, Social climate, Personal development via work and Work centrality) measuring psychosocial environment and 3 subscales covering mental burnout/fatigue, psychological stress and psychosomatic symptoms.

### Statistics

Due to a general deviation from the requirements of normality of most variables, non-parametric univariate statistics (Mann-Whitney, Kruskal-Wallis) were used for univariate analyses, and Classification Tree for multivariate analyses. When there are many potential explanatory variables Classification Trees (CT) can give a clear picture of the structure of the data and interaction among the variables [73]. Initially, all the explanatory variables are put together, and by examining one variable at a time, the algorithm then systematically splits them into - in the present case - two homogenous groups. The process is continued on each of the branches and the object is to attain as homogeneous a set of subgroups as possible in each partition until no more data splits can be found. In the case of categorical dependent variables, as in the present study, the model improvement is based on the homogeneity of the data within new nodes compared to those that were dichotomized. In contrast to the simultaneous decisions of discriminant analyses, Classification Tree is built on hierarchical evaluations and the final results can be summarized in a series of logical if-then conditions. There is no implicit assumption that the relationships between the predictor variables and the dependent variable should be linear, follow some specific non-linear function, or be monotonic.

In the tree-analyses, the variables from MBI, MHPSS, and PWSQ with relevance for educational (professional self-doubt, opportunities for personal development at work, relations to other professions), clinical (work pressure, work demands), and organizational sources (control over resources, influence and co-determination) sources of stress, in addition to variables measuring burnout and health and well-being (table 3) were used. In addition, a selection of variables from the PWSQ (basic demographics such as sex, age, education, employment, having been a recipient of violence, stress training, training in dealing with violence and support for stress) were utilized. The classification tree was estimated using the CRT routine in the SPSS. Data analyses were performed on the SPSS 16.00.

**Table 3:** Univariate differences in mean values between inpatient and community staff. Number of items in parenthesis. Mann-Whitney

		Variable definitions	Nurses	Unqualified	
MBI	Emotional exhaustion	Feelings of being emotionally overextended and exhausted	15.6 (8.9)	15.7 (10.1)	- .078; p = .94
	Depersonalization	Unfeeling and impersonal responses towards recipients of services	3.6 (4.7)	5.5 (5.7)	- 1.63; p = .10
	Personal accomplishment	Feelings of competence and achievement in one's work	36.3 (7.4)	37.3 (7.8)	- 1.11; p = .27

MHPSS^a)^	Workload	Too much work work, different tasks, lack of time, too long hours (6)	1.05 (.70)	.94 (.61)	- .848; p = .40
	Client related difficulties	Dealing with suffering, small improvement, demanding clients, threats (6)	1.01 (.56)	.89 (.59)	- 1.52; p = .13
	Organizational structure and processes	Lack of support from managers, poor management, organizational problems, structure and policies (6)	1.02 (.78)	1.01 (.79)	- .124; p = .90
	Relationships and conflicts with other professionals	Conflicts with other professions, conflicting roles, criticism, multidisciplinary teams, difficult colleagues (6)	.62 (.55)	.75 (.64)	- 1.14; p = .25
	Lack of resources	Inadequate staffing, physical environment, training opportunities (6)	1.12 (.62)	1.02 (.73)	- 1.53; p = .18
	Professional self doubt	Inadequately skilled, own capabilities, doubt about therapeutic effectiveness, keeping up to date, fear of making mistakes (6)	.96 (.59)	.98 (.70)	- .164; p = .87
	Home-work conflict	Not enough time with family, personal vs professional role (6)	.62 (.58)	.61 (.51)	- .232; p = .88
	MHPSS total	Overall stress scores based on above factors (6)	.91 (.46)	.88 (.52)	- .335; p = .74

Agervold	Physical environment	Noise, temperature, air, general quality (5)	1.70 (1.50)	1.54 (1.29)	- .455; p = .65
	Social relations^1)^	Cliques, conflict, quarrelling, lack of agreement (5)	.87 (.9)	1.21 (1.4)	- 1.91; p = .05
	Work pressure	Being busy, breaks, fulfil tasks, has to take work home (4)	1.88 (1.04)	1.80 (1.04)	- .537; p = .59
	Work demands ^2)^	Concentration, recalls, task difficulty, accessible solutions,	3.39 (.90)	2.88 (1.17)	- 3.25; p = .001
	Control of your work	Influence of own work, pace, autonomy, planning (4)	1.01 (1.29)	1.24 (1.25)	- 1.59; p = .10
	Influence and co-determination^3)^	Influence with regard to changes, organisation of the work, economy, strategies, allocation of resources (5)	2.04 (1.51)	2.46 (1.52)	- 1.76; p = .08
	Management style	Favouritism, criticism, inaccessible management, access to leaders (5)	1.24 (1.55)	1.25 (1.49)	- .210; p = .83
	Work role	Quality of rules, clear/unclear instructions, information, (5)	1.47 (1.38)	1.33 (1.37)	- .760; p = .45
	Personal development at work	Developing own capabilities, personal development, learning (5)	.35 (.82)	.74 (1.34)	- 1.63; p = .10
	Contact	Contact with colleagues during work, cooperation, social relations (5)	.86 (1.21)	.86 (1.05)	-. 633; p = .86
	Work orientation and motivation	Stimulating work, importance of salary, proud of the work (5)	.91 (1.00)	.99 (1.25)	- .180; p = .86
	Acting possibilities ^b)^	Change bad working conditions, help and support from the management and from colleagues (3)	3.30 (1.55)	3.15 (1.72)	- .556; p = .58
	Health and well-being Work) ^c)^	Need to relax, concerns, problems due to incompetence, motivation (5)	2.43 (1.11)	2.37 (1.08)	- .233; p = .82
	Health and well-being General ^d)^	(Restlessness, irritation, depressiveness, vertigo, heart beat, pains; seldom to almost daily (10)	4.32 (3.72)	4.08 (3.15)	- .157; p = .88
	Bullying	Criticism, ridiculing, being excluded, rumours (12)	.83 (1.60)	1.28 (2.33)	- 1.01; p = .31

^1) ^Z - 1.91, p = .06

^2) ^Z - 3.25, p = .001

^3) ^Z - 1.76 p = .08

^a) ^(No = 0/yes = 1)

^b) ^(4-point scale from "Bad" to "Very good")

^c) ^(No = 0/yes = 1)

^d) ^(4-point scale from "Never/seldom" to "Almost every day")

### Ethical issues

The national ethical committees relevant for each participating centre approved the study.

## Results

Qualified staff worked more hours per week, and had more often been recipients of violence from patients. N-R C staff was older, and were predominantly male.

### Univariate analyses

Table 3 shows the differences between the two study groups on the Maslach Burnout Inventory and the PWSQ. Neither the MBI nor the MHPSS subscales demonstrated significant differences between the two groups. The only significant differences were found on the PWSQ: N-R C staff were more dissatisfied with their social relations (p = .05) and qualified staff scored significantly higher on the work demand sub-scale of the MBI (p = .001).

### Multivariate analyses

**Figure 1:**
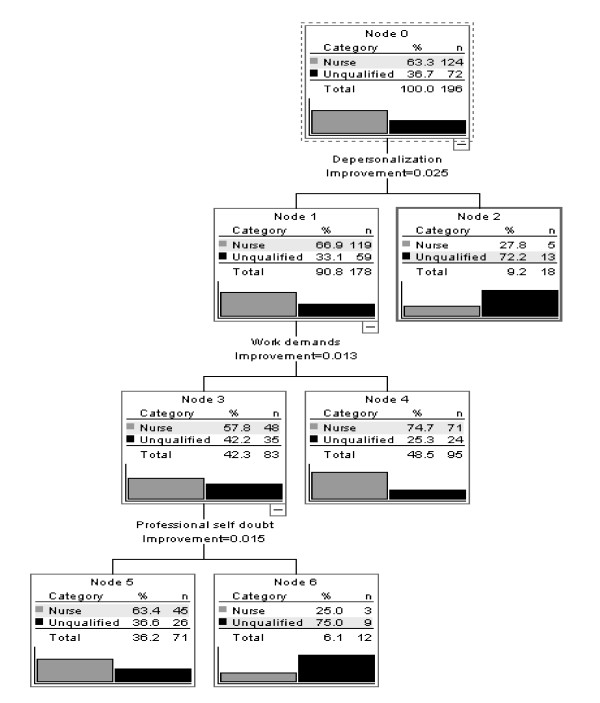
**Classification tree**. Nurses vs unqualified (N-RC) staff.

In addition to the selection of variables from the MBI, MHPSS and PWSQ, demographic variables from the ODQ (age, sex, marital status, education, other employments, hrs work per week, night work, having been a recipient of violence, and training in dealing with violence and support for stress) were used as independent variables for analysis. Figure 1 shows the results of this analysis.

Three groups with extreme scores on the stress- and burnout variables were identified. The first group was a numerically small group (N = 18) with high scores on the MBI Depersonalization variable. N-R C staff was strongly overrepresented in this group (N = 13 vs. N = 5).

The second group was a numerically large group (N = 95) characterized by low scores on the MBI Depersonalization SUBSCALE and high scores on the MBI Work Demands subscale. Qualified staff was overrepresented here (74.7 %). The third group had low scores on the MBI Depersonalization and Work Demands subscales. These were split according to their scores on the MHPSS subscale Professional Self-doubt. A small group of high self-doubters was identified. It consisted of a majority of N-R C staff (N = 9 vs. N = 3). The percentages of correct classification were 93.5 % for the nurses, but only 30.6 % of the N-R C staff.

### Univariate analyses of the three extreme groups

We performed some univariate analyses (chi square and Mann-Whitney) in an attempt to characterize those with high scores on Depersonalization, Low Depersonalisation and high Work Demand and low Depersonalisation/Work Demand and high Professional Self-doubt. We did this by comparing them with - as far as possible - similar-sized group on the same level with low score on the same variables (Cramer's V, Mann-Whitney).

We found that compared to the their low-depersonalisation colleagues, depersonalization high-scorers tended to be younger (34.9 vs. 43.1 yrs, p = .0025), males (p = .07), more of them had been recipients of violence from patients (95% vs. 69%, p = .026), they scored higher on Emotional Exhaustion (MBI) (23.8 vs. 11.00, p = .000) and had lower Personal Accomplishment score (MBI) (35.5 vs. 38.0, p = .001). One centre had 60 % of their staff in the high Depersonalisation group.

Compared with the low Depersonalisation/low Work Demand group, the low Depersonalisation/high Work Demand group had more often been recipients of violence (78.3% vs. 59.5 %. p = . 07) and had higher scores on Emotional Exhaustion (48.2 vs. 34.2, p = .000).

Extreme Professional Self-doubt was also context related as one of the centres had 5 of the 12 persons in the high self-doubt group (p = .051). Other differences were non-significant.

## Discussion

The assumption behind the study was that the sources of stress would be different between qualified and N-R C nursing staff, and that this would lead to N-R C staff being more prone to burnout than their formally better qualified colleagues. In both the univariate and multivariate analyses we found few and generally small differences between the two groups with regard to both sources of stress and burnout.

This was confirmed with respect to two of the stress-source variables, where the differences were univariately significant at the .05 level: N-R C staff experienced their social relations at work to be inferior (cliques, conflict, quarrelling, lack of agreement) compared the qualified staff. The qualified staff, on the other hand, complained more about high work demands (in terms of having to concentrate, task difficulty, availability of solutions etc).

The burnout subscales did not discriminate between the qualified and N-R C staff. This means that the hypotheses that guided the study (different sources of stress and different levels of burnout) only to a very limited extent (two out of 22 sources of stress, no differences in burnout) were confirmed. Also Jenkins' and Elliot's [31] study of stressors and burnout among qualified and N-R C staff in acute mental health services showed that the two groups did not differ much in terms of burnout, but that their sources of stress were to some extent different (adequate staffing and dealing with threatening and difficult patients).

Compared to the MBI norms[66], the percentages of qualified and N-R C staff that exceeded the normative values were on Emotional Exhaustion (High ≥ 27, Average 17 - 26 and Low ≤ 16) were 8.9 % vs. 12.5 %), on Depersonalisation (≥ 13, 7-12 and ≤ 6, resp.) .8 % vs. 9.7 %, and on Personal Accomplishment (≥ 39, ≥ 32-38 and ≤ 31 resp.) 25.4 % vs. 20.8 %. Thus, in terms of extreme scores, the differences between the two groups were greatest on Depersonalization. High burnout scores did not in general characterise the two groups. Personal Accomplishment was an area where a substantial proportion of both groups had high scores and here high score signifies feelings of achievement. A positive self-image may counteract burnout [74] and feelings of Personal Accomplishment may help to explain the rather low average value on emotional exhaustion and depersonalisation.

On most of the stress source measuring variables the absolute scores were low which implied that neither groups were particularly dissatisfied or strained. There were some exceptions, most strikingly on the variables Work Load (too much work, many different tasks, lack of time, too long hours), Work Demands (having to concentrate, lots of things to recall, task difficulty, accessible solutions), Influence and co-determination (influence on organisational changes, organisation of the work, economy, strategies, allocation of resource), Acting Possibilities (be able to change bad working conditions, receive help and support from the management and from colleagues), Work Related Health (need to relax, concerns, problems due to incompetence, motivation etc.) and Lack of Resources (Inadequate staffing, physical environment, training opportunities). On all these variables both groups had high ratings. This suggests a common experience among both the qualified and the N-R C groups of staff - in spite of low average burnout and high personal accomplishment scores - of having limited influence on a work situation characterised by clinical pressure and insufficient resources. Similar results are reported in other studies of mainly nurses [22,55,75,47,76].

The choice of classification tree as the multivariate statistics made identification of subgroups possible. The analyses identified three groups with (to some extent) extreme negative ratings:

(i) a numerically small group (a) (N = 18) with high scores on the MBI depersonalization variable. N-R C staff was strongly overrepresented. When the high-Depersonalisation group was compared to a random similar-sized group of staff with the lowest ratings on this variable, univariate analyses showed that high depersonalization-scorers were younger, more of them lacked professional qualifications of any sort and the majority came from one of the centres. One centre had 60 % of their participators in the high depersonalisation group. Depersonalization is the interpersonal aspect of the burn-out syndrome [77] and refers to uncaring and impersonal attitudes towards care or service recipients. As mentioned, we also found that a higher proportion (9.7 %) of N-R C staff exceeded the normative values for high scores of the MBI Depersonalization-norms, but in terms of persons the number is only 7, which highlights the centre-specific character of the problem. Depersonalisation usually occurs among overburdened personnel who receive little positive feedback/rewards [50]. A study by Hare and Pratt found high levels of emotional exhaustion and depersonalisation among nursing assistants [78]. According to Kanste et al [79] leadership that encourages the employees to look beyond their own self-interest for the good of the group (so-called transformational leadership) seem to protect from depersonalization.

(ii) Qualified staff was overrepresented in a numerically large group that was low on Depersonalization and high on Work Demands. The Work Demand variable measures concentration, keeping many things in mind, task difficulty and having to deal with situations where solutions may be difficult to find). Nurses perceive stressors differently according to their grade [80], and the obvious explanation for the high Work Demand score is that qualified staff more often have managerial positions and have more responsibility for clinical decisions. Comparing random samples of this group with colleagues who scored low on depersonalisation and on Work Demands, univariate analyses showed that the low depersonalisation/high Work Demand group had more often been recipients of violence at work and had higher scores on Emotional Exhaustion. Other studies have found correlations between high work load and Emotional Exhaustion [31,81], but the Work Demand variable in the present study measures other characteristics of work than work load. Our Work Load variable showed no significant differences between the two groups.

(iii). A second small group (N = 12) also with a majority of N-R C staff had high scores on Professional Self-doubt, but were low on Work Demands and on Depersonalization. Professional Self-doubt is a MHPSS subscale that measures feelings of being inadequately skilled, uncertainty about own capabilities, doubting the efficacy of therapeutic endeavours and fear of making mistakes in the treatment. As with the high depersonalization group, extreme Professional Self-doubt was context related: 41% of this group came from one centre.

The traditional rigid workforce model is non-functional in complex interactional systems, not the least in community-based services. Thornley [29] found that N-R Cs through experiential learning acquired substantial clinical competencies that were useful in a variety of different roles and she argues for a comprehensive re-evaluation of the competencies of non-registered caregivers. N-R C's position within the health system is said to be both central and marginal: the services depend upon them at the same time as N-R Cs are often considered to be substitutions for more valued staff. The uncertainty often surrounding the N-R C's position may reduce the interest in important professional issues such as training, career, regulation and inclusion of these persons into the health services. This may result in a situation where non-registered personnel are less appreciated because they are insufficiently trained, and - as a result - are insufficiently trained because they are not well valued. Whereas qualified personnel are responsible for the duties within their profession, non-registered personnel perform only functions that are delegated to them, and they work under the supervision and guidance of fully qualified personnel [82]. This can result in more work-related strain, more burnout problems and less stable services.

Our results showed only small differences in burnout and sources of stress between N-R Cs and nurses. The main implication of the study is that no general measures addressed towards the N-R Cs with regard to stress and burnouts seem necessary. This has consequences for recruiting and retaining this staff group. However, the study also showed that centre-specific problems may cause more problems among N-R Cs than among the qualified staff. When N-R Cs from one of the centres reported high score on Professional self-doubt, this gave limited support to one of the hypothesis behind the study, namely that lacking formal qualifications in a highly specialised field might be experienced as stressful, and - when relevant - requires to be dealt with.

Both groups reported a limited influence on a work situation characterised by clinical pressure and insufficient resources. This means that alleviating measures should reach both groups. As we have argued above, the mental health services will continue to depend on less formally qualified staff and including these in the work force will require the development of special career-pathways, an example being the UK initiatives (NIMHE, 2005 #210}. A study by Caudhill and Patrick [83] found that nursing assistants who were planning to leave their present jobs were younger, had been in their positions shorter, were paid less, ranked their nursing skills lower and were better educated than those who planned to stay. At least some of these factors can be met by more systematic career-planning. Better trained and educated N-R Cs can also to some extent alleviate the significantly higher Work load reported by qualified nurses. Wages, fringe benefits, job security, and alternative choices of employment are important determinants of job tenure that should be addressed, in addition to training and organizational culture [84].

### Limitations of Study

It was not possible to ensure that the participating countries were randomly drawn from all EU countries, nor that therefore their services were in any sense necessarily representative of the EU as a whole.

Thus it is difficult to generalise as to what degree any of the centres were representative of typical working conditions operative in their own countries on a wider basis.

Whilst the MBI already was translated reliably into all the languages pertinent to this study, this was not the case for the ODQ, PWSQ or the MHPSS. While it was anticipated that back translation would occur during the course of the project, the complexity and cost of the task could not be born by the project's funding. However, all of the key researchers were English speakers as a second or third language and in one site, Bodo (Norway), the researcher was a native English speaker. Consideration must also be given to the sample sizes achieved for each site and team type.

## Conclusions

There were few univariate differences in the sources of stress and levels of burnout among qualified and N-R C staff. N-R C staff more often reported negative social relations at work, lower Work Demands and were overrepresented among those who exceeded the above-threshold values on the Depersonalisation subscale. The last group were small and seemed to mainly reflect a centre-specific problem. (2) The highest scores for both groups were on stress source variables that measured feelings of not being able to influence a work situation characterised by great demands and insufficient resources. Procedures for dealing with stress should be made available on a routine basis. (3) High Work Demand was the variable that most clearly distinguished the two groups. It was more prevalent amongst qualified staff.

## Competing interests

The authors declare that they have no competing interests.

## Authors' contributions

KWS participated in the design of the project, analysed the data, drafted the manuscript. PR designed the project, helped to draft the manuscript. ID participated in the design of the study, collected data and helped to draft the manuscript. All authors read and approved the final manuscript. The OSCAR group participated in the planning and management of the study.

## Pre-publication history

The pre-publication history for this paper can be accessed here:

http://www.biomedcentral.com/1472-6963/10/163/prepub
